# Evaluation of the Standard Diagnostics *Leptospira* IgM ELISA for diagnosis of acute leptospirosis in Lao PDR

**DOI:** 10.1016/j.trstmh.2012.06.002

**Published:** 2012-09

**Authors:** Ampai Tanganuchitcharnchai, Lee Smythe, Michael Dohnt, Rudy Hartskeerl, Manivanh Vongsouvath, Viengmone Davong, Olay Lattana, Paul N. Newton, Stuart D. Blacksell

**Affiliations:** a Mahidol–Oxford Tropical Medicine Research Unit (MORU), Faculty of Tropical Medicine, Mahidol University, 420/6 Rajvithi Road, Bangkok 10400, Thailand; b WHO/FAO/OIE Collaborating Centre for Reference & Research on Leptospirosis, Queensland Health Forensic and Scientific Services, Coopers Plains, Brisbane, Queensland 4108, Australia; c WHO/FAO/OIE Collaborating Centre for Reference and Research on Leptospirosis, KIT Biomedical Research, Meibergdreef 39, 1105 AZ Amsterdam, The Netherlands; d Lao-Oxford-Mahosot Hospital-Wellcome Trust Research Unit, Mahosot Hospital, Vientiane, Lao PDR; e Centre for Clinical Vaccinology and Tropical Medicine, University of Oxford, Churchill Hospital, Oxford OX3 7LJ, UK

**Keywords:** *Leptospira*, ELISA, Diagnosis, Cut-off

## Abstract

The diagnostic utility of the Standard Diagnostics *Leptospira* IgM ELISA for detection of acute leptospirosis was assessed in febrile adults admitted in Vientiane, Laos. Using the cut-off suggested by the manufacturer [optical density (OD) ≥0.75], the assay demonstrated limited diagnostic capacity with a sensitivity of 95% and a specificity of 41% compared with the *Leptospira* microscopic agglutination test, which is the serological gold standard. However, re-evaluation of the diagnostic cut-off to an OD of 1.7 demonstrated improved diagnostic accuracy overall (sensitivity 70%; specificity 78%).

## 1 Introduction

Laboratory diagnosis of acute *Leptospira* infection in endemic settings is problematic as there is a paucity of simple, inexpensive, well characterised assays that are diagnostically informative. The serological ‘gold standard’ is the microscopic agglutination test (MAT), which requires paired specimens and considerable technical resources and training and, in some cases, is not useful for acute patient management.^1^ Simple IgM antibody detection-based ELISAs are marketed as being accurate for the diagnosis of *Leptospira* infection, however their accuracy is dependent on the background immunity of the local population and the number of days of illness.^2^ Here we report the evaluation of a commercial ELISA for the detection of *Leptospira* IgM antibodies among adults with fever in the leptospirosis-endemic setting of the Lao People's Democratic Republic (Laos) to determine (i) its utility for diagnosis of acute leptospirosis and (ii) a locally appropriate diagnostic cut-off.

## 2 Materials and methods

Human serum was collected as part of a previously described study^2^ following informed consent as part of a study to determine the causes of unexplained fever in patients presenting at Mahosot Hospital (Vientiane, Laos) between November 2001 and October 2003.^3^ Admission (n = 184) and convalescent (n = 151) sera were collected from 184 patients (total samples, n = 335) and were stored at –20 °C until tested. Ethical approval was granted by the Ethical Review Committee of the Faculty of Medical Sciences, National University of Laos, Vientiane, Laos.

A commercial ELISA (Standard Diagnostics, Yongin-si, South Korea) for the detection of IgM antibodies against *Leptospira* spp. was performed according to the manufacturer's instructions at Mahidol University–Oxford Tropical Medicine Research Unit in Bangkok, Thailand. An optical density (OD) of ≥0.75 was defined as positive according to the manufacturer's method. Reference serology methodologies have been described elsewhere.^2^ The MAT for *Leptospira* antibodies was performed by the WHO/FAO/OIE Collaborating Centres for Reference & Research on Leptospirosis at KIT Biomedical Research (Amsterdam, The Netherlands) and in Brisbane (Australia). Using MAT diagnostic criteria, a patient was considered to have an acute or recent *Leptospira* infection if a serum showed a MAT titre of ≥1:400 or demonstrated a ≥4-fold rise in MAT titre against the same serovar between admission and convalescent serum samples. Diagnostic accuracy was calculated for the ELISA by comparing results with the acute and convalescent MAT results for each patient as an individual case diagnosis. Standard diagnostic accuracy indices of sensitivity, specificity, negative predictive value and positive predictive value with exact 95% CIs as well as IQR of days of fever and area under the receiver operator characteristic (ROC) curves (AUROCC) were calculated using Stata/SE 10.0 (StataCorp LP, College Station, TX, USA).

## 3 Results and discussion

The percentage of patients with a true leptospirosis infection (as defined by MAT diagnostic criteria) was 12.5% (23/184), of which 12 had a ≥4-fold rise in titre between admission and convalescent samples. On admission, patients had been ill for a median of 9 days (IQR 7–13 days) and the median interval between admission and convalescent sera was 4.5 days (IQR 2–8 days). Using the manufacturer's suggested cut-off of an OD of 0.75, diagnostic sensitivity for acute diagnosis was high (90–96%) (Table 1), however specificity was generally poor with a significantly lower specificity for convalescent sera than for admission sera (convalescent 28% vs admission 53%; Pearson's χ^2^ = 34.471; p≤0.0005), which may be explained by the large number of convalescent samples that demonstrated a non-specific rise in the OD to beyond the 0.75 cut-off. Samples from patients with only 1–7 days of fever had higher specificity (72%) but with very wide confidence intervals (Table 1). AUROCC analysis of ELISA accuracy versus MAT results gave an AUROCC of 0.82 (95% CI 0.75–0.89), suggesting that the ELISA was marginally informative. Modelling of positivity cut-off values to improve the accuracy of the ELISA (using ROC curve analysis) demonstrated that by increasing the positivity cut-off to values approximating 1.5 gave a compromise between sensitivity (70–73%) and specificity (69–78%) that provided marginally sufficient accuracy for diagnostic utility. Examination of diagnostic accuracy for the 1–7-day fever samples using the positivity cut-off values in the 1.4–1.7 OD range, the sensitivity was 80% and specificity ranged from 82% to 87% (Table 1), which may be accurate to find application for the diagnosis of acute *Leptospira* infection.

**Table 1:** Sensitivity and specificity (with 95% CIs) of the Standard Diagnostics *Leptospira* IgM ELISA for the detection of IgM antibodies compared with the microscopic agglutination test (MAT)

Cut-off OD	Sample timing	Days since fever onset [median (IQR)]	ELISA result	MAT (n)		Sensitivity (%)	Specificity (%)
				+	–		
0.75	Overall	10 (7–14.5)	+	38	173	95.0 (83.2–99.4)	41.4 (35.7–47.2)
			–	2	122		
	Admission	9 (7–13)	+	22	76	95.7 (78.1–99.9)	52.8 (44.8–60.7)
			–	1	85		
	Convalescent	15 (10–20)	+	16	97	94.1 (71.3–99.9)	27.6 (20.2–36.0)
			–	1	37		
	Days 1–7 of fever^a^	6 (5–7)	+	9	17	90.0 (55.5–99.7)	71.7 (58.6–82.5)
			–	1	43		
1.4	Overall		+	29	91	72.5 (56.1–85.4)	69.2 (63.5–74.4)
			–	11	204		
	Admission		+	18	38	78.3 (56.3–92.5)	76.4 (69.1–82.7)
			–	5	123		
	Convalescent		+	11	53	64.7 (38.3–85.8)	60.4 (51.6–68.8)
			–	6	81		
	Days 1–7 of fever^a^		+	8	11	80.0 (44.4–97.5)	81.7 (69.6–90.5)
			–	2	49		
1.5	Overall		+	29	81	72.5 (56.1–85.4)	72.5 (67.1–77.6)
			–	11	214		
	Admission		+	18	36	78.3 (56.3–92.5)	77.6 (70.4–83.8)
			–	5	125		
	Convalescent		+	11	45	64.7 (38.3–85.8)	66.4 (57.8–74.3)
			–	6	89		
	Days 1–7 of fever^a^		+	8	10	80.0 (44.4–97.5)	83.3 (71.5–91.7)
			–	2	50		
1.6	Overall		+	29	71	72.5 (56.1–85.4)	75.9 (70.6–80.7)
			–	11	224		
	Admission		+	18	33	78.3 (56.3–92.5)	79.5 (72.4–85.5)
			–	5	128		
	Convalescent		+	11	38	64.7 (38.3–85.8)	71.6 (63.2–79.1)
			–	6	96		
	Days 1–7 of fever^a^		+	8	9	80.0 (44.4–97.5)	85.0 (73.4–92.9)
			–	2	51		
1.7	Overall		+	28	64	70.0 (53.5–83.4)	78.3 (73.2–82.9)
			–	12	231		
	Admission		+	17	30	73.9 (51.6–89.8)	81.4 (74.5–87.1)
			–	6	131		
	Convalescent		+	11	34	64.7 (38.3–85.8)	74.6 (66.4–81.7)
			–	6	100		
	Days 1–7 of fever^a^		+	8	8	80.0 (44.4–97.5)	86.7 (75.4–94.1)
			–	2	52		

OD: optical density.

KIT panel of strains with serovars in parentheses: Jez Bratislava (Bratislava); Mus 127 (Ballum); Hond Utrecht IV (Canicola); Duyster (Grippotyphosa); Mandemakers (Grippotyphosa); Hebdomadis (Hebdomadis); Kantorowicz (Icterohaemorrhagiae); Wijnberg (Copenhageni); Poi (Poi); Pomona (Pomona); 1161 U (Proechimys); Hardjoprajitno (Hardjo); Lely 607 (Hardjo); Mus 24 (Saxkoebing); M 84 (Sejroe); Patoc I (Patoc); CH 11 (Andamana); Ballico (Australis); Rachmat (Rachmati); Swart (Bataviae); Celledoni (Celledoni); 3522 C (Cynopteri); Sari (Mini); CZ 214 K (Panama); Salinem (Pyrogenes); Veltat Semarang 173 (Semaranga); 1342 K (Shermani); and Perepelitsin (Tarassovi).

Australian panel of serovars: Pomona; Hardjo; Tarassovi; Grippotyphosa; Celledoni; Copenhageni; Australis; Pyrogenes; Canicola; Hebdomadis; Mini; Sarmin; Autumnalis; Cynopteri; Ballum; Bataviae; Djasiman; Javanica; Panama; Shermani; and Mwalok.

a Combination of admission and convalescent specimens.

Defining a diagnostic cut-off for an antibody-based assay in a *Leptospira*-endemic setting is a compromise between specificity and sensitivity. The persistence of anti-*Leptospira* IgM antibodies for many months following recovery from leptospirosis and repeated exposure to non-pathogenic *Leptospira* during farming^4^ may explain the poor specificity (false positivity) of antibody-based assays for acute diagnosis.^5^ Because of the relatively short interval (median 4.5 days) between admission and convalescent sample collection (ideal minimum would be 7 days), we have used a single high MAT titre of ≥1:400 to define an acute infection in addition to using rising MAT titres. This may account for some additional false positivity owing to persistence of IgM antibodies following previous infections. Similar observation of poor specificity of an anti-*Leptospira* IgM rapid assay was reported in Vietnam where a high proportion of clinically well individuals gave positive IgM results.^5^

This study suggests that the diagnostic accuracy of this ELISA for diagnosis of acute leptospirosis in Laos is improved when the diagnostic cut-off is optimised using ROC curve analysis compared with that provided in the manufacturer's instructions. Further studies are required to determine the utility of this assay for acute diagnosis and epidemiology in other leptospirosis-endemic and non-endemic settings as it is likely that such ‘tuning’ of ELISA cut-offs is needed in different epidemiological settings. Further studies are also required to determine the diagnostic utility of this and other such assays as simply antibody detection tools for application in epidemiological surveillance. There is a clear need for implementation and local validation of new serological assays and PCRs for acute diagnosis of leptospirosis infection.

## Authors’ contributions

PNN, SDB and AT conceived and designed the study; SDB, AT, MV, VD, OL, LS, RH and MD analysed and interpreted the data; SDB, AT and PNN drafted the manuscript. All authors critically revised the manuscript for intellectual content and read and approved the final version. SDB and PNN are guarantors of the paper.

## Funding

Wellcome Trust of Great Britain; Embassy Small Grants Scheme.

## Competing interests

None declared. The ELISA tests were provided without charge by Standard Diagnostics (Yongin-si, South Korea). Standard Diagnostics had no role in the design, execution, analysis, writing or submission of this paper.

## Ethical approval

Ethical approval was granted by the Ethical Review Committee of the Faculty of Medical Sciences, National University of Laos, Vientiane, Laos.
